# Supplementary material for: South Asian origin and global transmission history of Mycobacterium tuberculosis lineage 4
Source: mSystems. 2025 May 20;10(6):e00427-25. doi: 10.1128/msystems.00427-25 (PMC12172454; doi:10.1128/msystems.00427-25)
Supplement: Supplemental material — Supplemental methods and Figures S1 to S6. [file msystems.00427-25-s0001.docx]

# South Asian origin and global transmission history of *Mycobacterium tuberculosis* lineage 4

**Running title**

Global transmission history of *M. tuberculosis* L4

**Authors**

Bharkbhoom Jaemsai^1,2^, Prasit Palittapongarnpim^1,2^, Pakorn Aiewsakun^1,2^*

**Author affiliations**

^1^ Department of Microbiology, Faculty of Science, Mahidol University, Bangkok, Thailand

^2^ Pornchai Matangkasombut Center for Microbial Genomics, Department of Microbiology, Faculty of Science, Mahidol University, Bangkok, Thailand

* **Corresponding author**

Pakorn Aiewsakun: Department of Microbiology, Faculty of Science, Mahidol University, 272, Rama VI Road, Ratchathewi, Bangkok, 10400, Thailand; +66 2 201 5676; pakorn.aie@mahidol.ac.th; ORCiD: 0000-0002-5665-4041

# Supplementary methods

## Compilation of WGS data of contemporary samples

We compiled 12,496 accession numbers of whole-genome short-read sequences of contemporary MTB L4 samples reported worldwide from research publications and public repositories (NCBI’ SRA and EBI-EMBL’ ENA). Metadata were retrieved from the ENA database using a modified version of the fetchngs pipeline (1). For samples whose metadata could not be obtained by this means, we attempted to retrieve their metadata directly from corresponding publications. Data compilation was performed up until August 2024. Samples with unknown geographical origins (n = 407) were excluded from our study.

We were able to retrieve sequence data for all samples, except two (n = 12,087). These data sets were first cleaned and mapped to the H37Rv reference genome (NCBI’s RefSeq accession number: NC_000962.3) using snpplet (https://github.com/CENMIG/snpplet) with default settings to quantify sequencing coverage and depth and to generate genomic variant calling format (GVCF) files. For samples with multiple sequencing data sets (identified by identical BioSample accession numbers), those with the highest mean read-mapping depth were retained (n = 34 excluded). Samples with overall read-mapping coverage below 90% and depths below 10× were also removed (n = 874), resulting in a clean data set of 11,179 high-quality contemporary samples. The GVCF files were subsequently analysed to assign taxonomic groups by using mtbtyper (https://github.com/ythaworn/mtbtyper), and determine drug resistance types by using TB-Profiler (2), both with default settings.

## Ancient samples

WGS data of three ancient MTB L4 samples were added to the data set, including LUND1 (accession number: SRR11524778), B80 (ERR651003), and B92 (ERR651004). Sequence reads were mapped to the H37Rv reference genome (NCBI’s RefSeq accession number: NC_000962.3) using Eager (3), and nucleotide variants were called. In the base calling, the parameter ‘udg_type’ was set to ‘full’ for LUND1, as its DNA library was a uracil DNA glycosylase library (4), and to ‘none’ for B80 and B92, as their libraries were not treated (5). The resulting GVCF files were analysed by mtbtyper (https://github.com/ythaworn/mtbtyper) and TB-Profiler (2) to assign taxonomic groups and to determine drug resistance types, respectively, both with default settings.

## Evolutionary rate and timescale estimation

The rate and timescale of MTB L4 evolution were estimated in two steps. First, we conducted a tip-dating analysis with BEAST v1.10.4 (6) on a down-sampled data set to best estimate the rate and timescale of the bacterial evolution under the best-fit tree and clock model, using only samples with relatively precise collection dates available (at least at the level of collection year). We then used the obtained rate estimate to time-calibrate the global MTB L4 tree together with tip sampling dates under a more scalable maximum likelihood (ML) framework, including samples with collection date ranges and those without collection dates.

### Initial Bayesian tip-dating analysis

To make the computation feasible, the initial Bayesian tip-dating analysis was conducted on a down-sampled data set. The data set was down-sampled by randomly pruning the global tree using Treemmer (7) with the option ‘prune_random’. The down-sampling was performed with the following criteria: i) retaining the three ancient samples in the down-sampling, ii) for each of the nine major sub-lineages and each of the unassigned sub-clades basal to them, sampling five sequences from each of the nine geographical regions, and iii) for lineage/region combinations that had fewer than five sequences, retaining all of them. This was to preserve as much temporal signal and genetic and geographic diversity as possible. This, indirectly, also reduced potential biases in the rate estimation potentially introduced by overrepresentation of samples from certain outbreaks with many isolates reported. This procedure resulted in a down-sampled data set of 377 sequences (**Table S2**). Tip dates of ancient samples were assumed to be their human host’s year of death, while sampling years were used for contemporary samples.

A multiple sequence alignment of the down-sampled sequences was then generated, and their ML phylogeny was estimated using the same procedure as described above. A root-to-tip regression analysis suggested that there was a sufficiently strong temporal signal in the data set to allow for Bayesian tip-dating and rate estimation (slope = 1.02×10^-7^ s/n/y, p value < 0.001). In addition, the data set was assessed for the TDRP by computing four evolutionary rate estimates over various timescales ranging between 28 to 340 years by using LSD2 (8) and examining if there was a significantly negative correlation between the rate estimates and the measurement timescales (9, 10). The rate estimations were performed with the option ‘-r a’ to automatically find the root placement on all branches, ‘-v 2’ to run the analysis twice, where in the second run, variances were calculated based on the estimated branch length of the first run, and ‘-f 1000’ to compute confidence intervals of rate and date estimates based on 1,000 simulated trees. The result suggested no significant evidence for the TDRP (slope = 0.005, p = 0.9673; **Fig S2**), justifying the standard tip-dating analysis.

Three tree prior models were examined the initial Bayesian tip-dating analysis: i) the constant population size tree model, ii) the Bayesian Skyride coalescent tree model, and iii) the Bayesian Skygrid tree model, each with two molecular clock models: i) the strict clock model and ii) the uncorrelated lognormal relaxed clock model. All analyses were done using the GTR+F+I nucleotide substitution model, identified as the best-fit model among all models supported by BEAST (6) as determined under the Bayesian information criterion with ModelFinder (11) implemented in IQ-TREE2 (12). For all analyses, the Markov Chain Monte Carlo (MCMC) sampling length was set to 1.0×10^9^ steps, and the parameter values were logged every 100,000^th^ step. BEAST XML files were manually edited to account for the number of bases in constant sites. Tracer (13) was used to inspect the results to ensure trace mixing and convergence. Effective sample size (ESS) values of all parameters were > 200 for all six analyses.

To determine the best-fit tree and clock models, their marginal likelihoods were computed using the path-sampling and stepping-stone methods, implemented in BEAST v1.10.4 (6) with 100 sampling paths. Each sampling path was run for 1.0×10^7^ steps, and the parameter values were logged every 1,000^th^ step. The marginal likelihood estimates were then compared to compute Bayes factors to identify the best-fit model (**Table S3**). The Bayesian maximum clade credibility phylogeny was then computed from the posterior tree distribution obtained under the best-fit model, discarding the first 10% of the MCMC sampling chain as burn-in, using TreeAnnotator utility in BEAST package (6).

To assess the robustness of our evolutionary rate and date estimates, the analysis was repeated with two independently down-sampled data sets under the best-fit prior settings (see **Table** **S2** for the lists of sequences used). Convergence and mixing were assessed by using Tracer (13), and ESS values of all parameters were > 200.

### ML tip-dating analysis of the global MTB L4 tree

We subsequently used the obtained rate estimate (1.07×10^-7^ s/n/y) to inform the tip-dating analysis of the global MTB L4 tree (n = 11,154) under an ML framework with LSD2 (8), which was much less computationally expensive. This tip-dating analysis also included samples with collection date ranges and those without collection dates, accounting for their collection date uncertainty. For sample with collection dates, collection years were used as their tip dates. For samples with collection date ranges, the reported year ranges were used. For samples without collection years, the upper bound of the collection time was set to the year when they were first made publicly available, without a lower-bound restriction. The analysis was performed with the option ‘-v 2 -f 1000’.

## Analysis of Sabin *et al.*’s L4 specific data set

Lastly, to further validate our results, we applied our data preparation and Bayesian tip-dating analysis protocol to the L4-specific data set analysed Sabin *et al.* (4) to estimate the rate and timescale of MTB L4 (n = 134, 131 contemporary samples, and 3 historical genomes, including LUND1: 1679 CE, B80: 1805 CE, B92: 1787 CE). The Bayesian Skygrid coalescent tree model, the strict molecular clock, and the GTR+F+I substitution model were used. Nevertheless, we found that, apart from the three historical genomes, all contemporary samples did not have collection dates. We therefore performed tip-date sampling for all contemporary genomes over a uniform distribution between 1992 and 2010, following the protocol used by Sabin *et al.* (4). MCMC sampling length was set to 1.0×10^9^ steps, and the parameter values were logged every 100,000^th^ step. The BEAST XML file was manually edited to account for the number of bases in constant sites. Tracer (13) was used to inspect the results to ensure trace mixing and convergence. ESS values of all parameters were > 200.

## Phylogeographic analysis

The geographical origin and global transmission history of MTB L4 was inferred through stochastic character mapping analysis using the global time-calibrated ML tree and the sampling locations of the samples. The analysis was performed with the phytools package (14) in R (15). Three models of geographical location state transition processes were fitted on the time-calibrated tree using the fitMk.parallel function from phytools (14): i) the equal-rates model (ER), ii) the symmetric-rates model (SYM), and iii) the all-rates-different model (ARD). Each model was evaluated with three state prior distributions on the root node: i) the “estimated” distribution, ii) the “empirical” distribution, and iii) the “FitzJohn” distribution. The ARD model with the FitzJohn root prior was identified as the best fit combination based on the Akaike Information Criterion (**Table S4**), and was therefore used in the final analysis. The analysis was conducted using the simmap function in phytools (14) with 1,000 simulations, each employing a state transition matrix sampled from the posterior distribution of the model (Q = “mcmc”).

# Supplementary figures


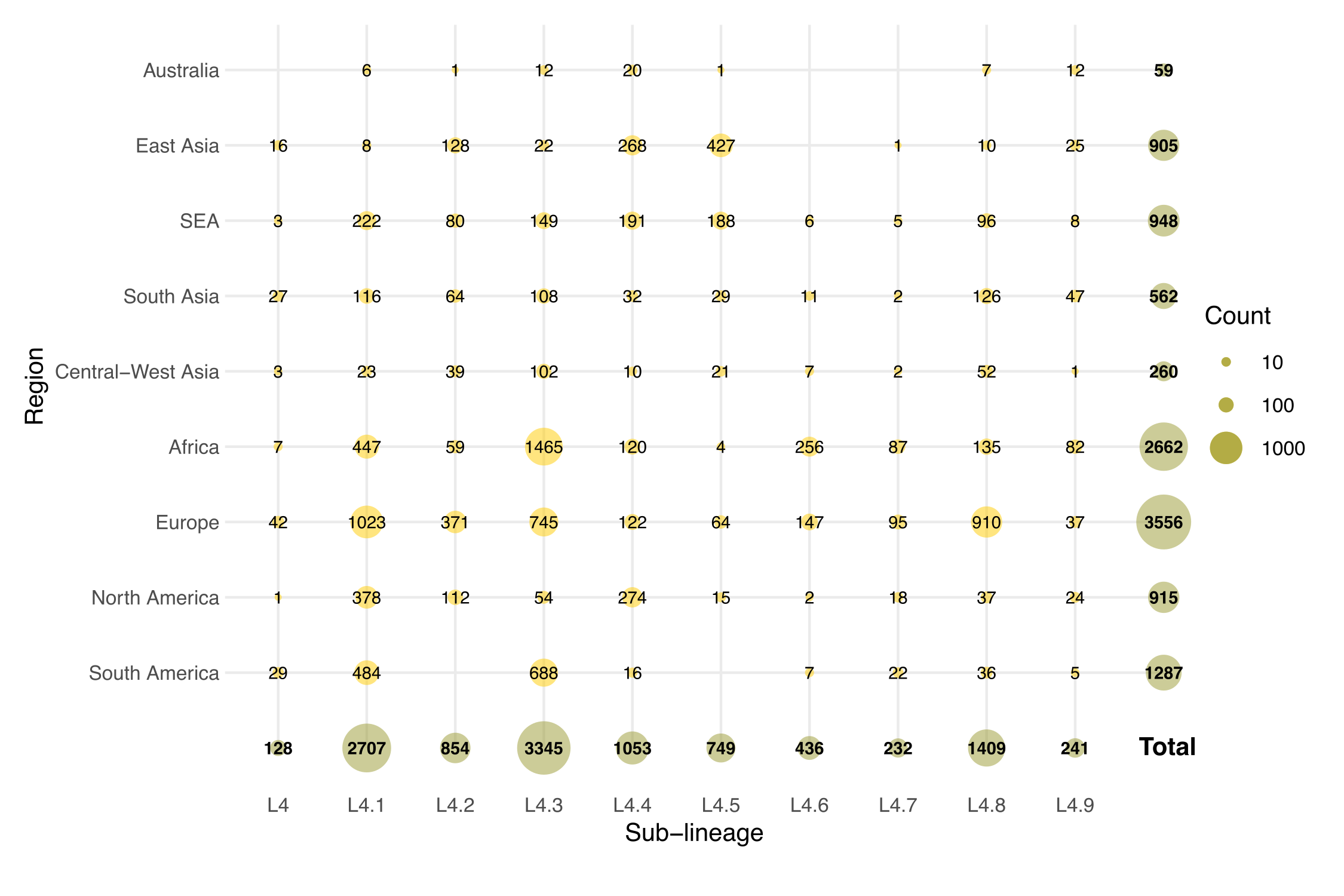


**Fig S1: Distribution of sequence samples by region and major sub-lineage.**


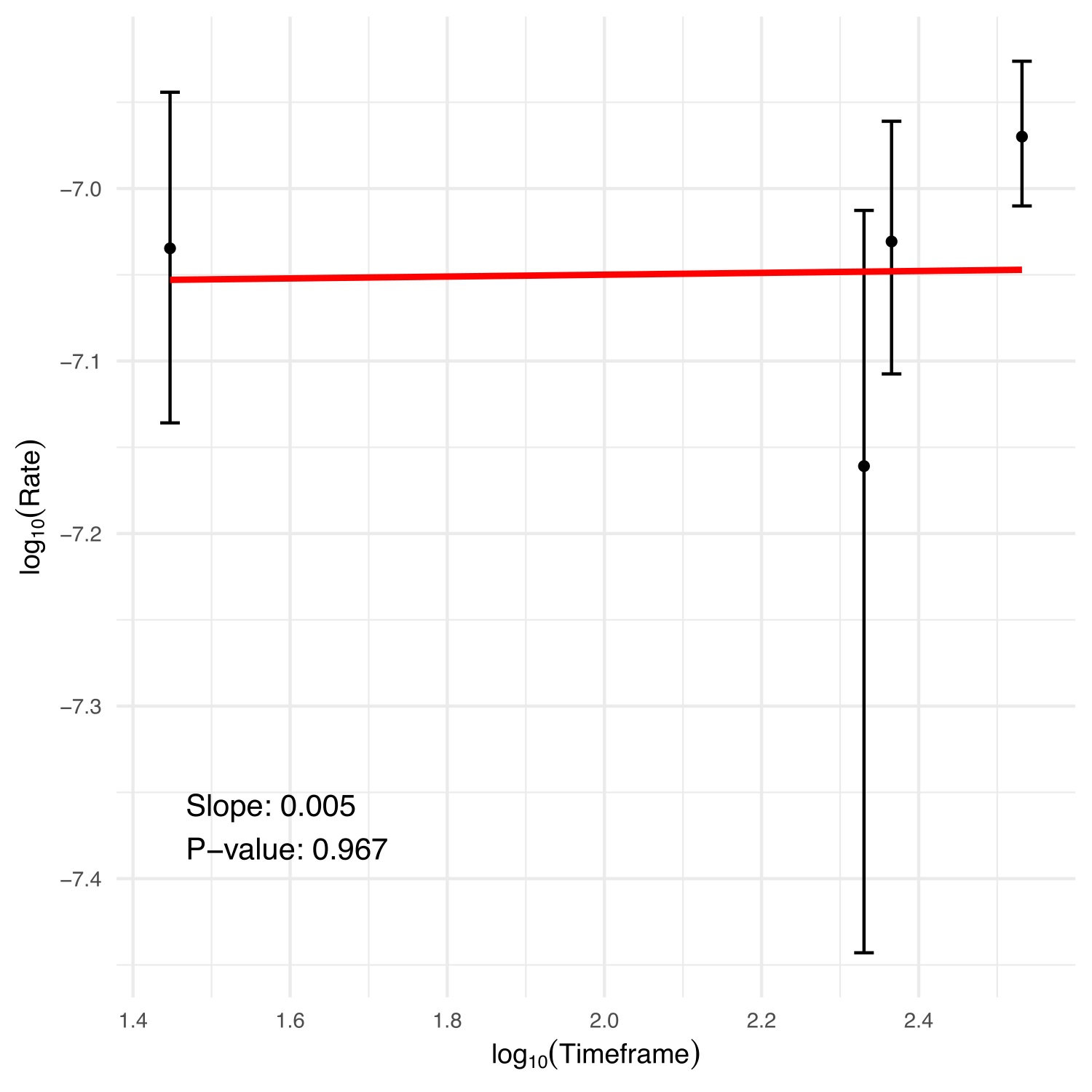


**Fig S2: Evolutionary rates estimated over four different timescales (ranging from 28 to 340 years).** A linear model fitted to log rate estimates and log measurement timescales is shown (red line) with its slope and corresponding p value. No significant correlation between rate estimate values and the timescales over which they were measured was detected (slope’s p value = 0.967).


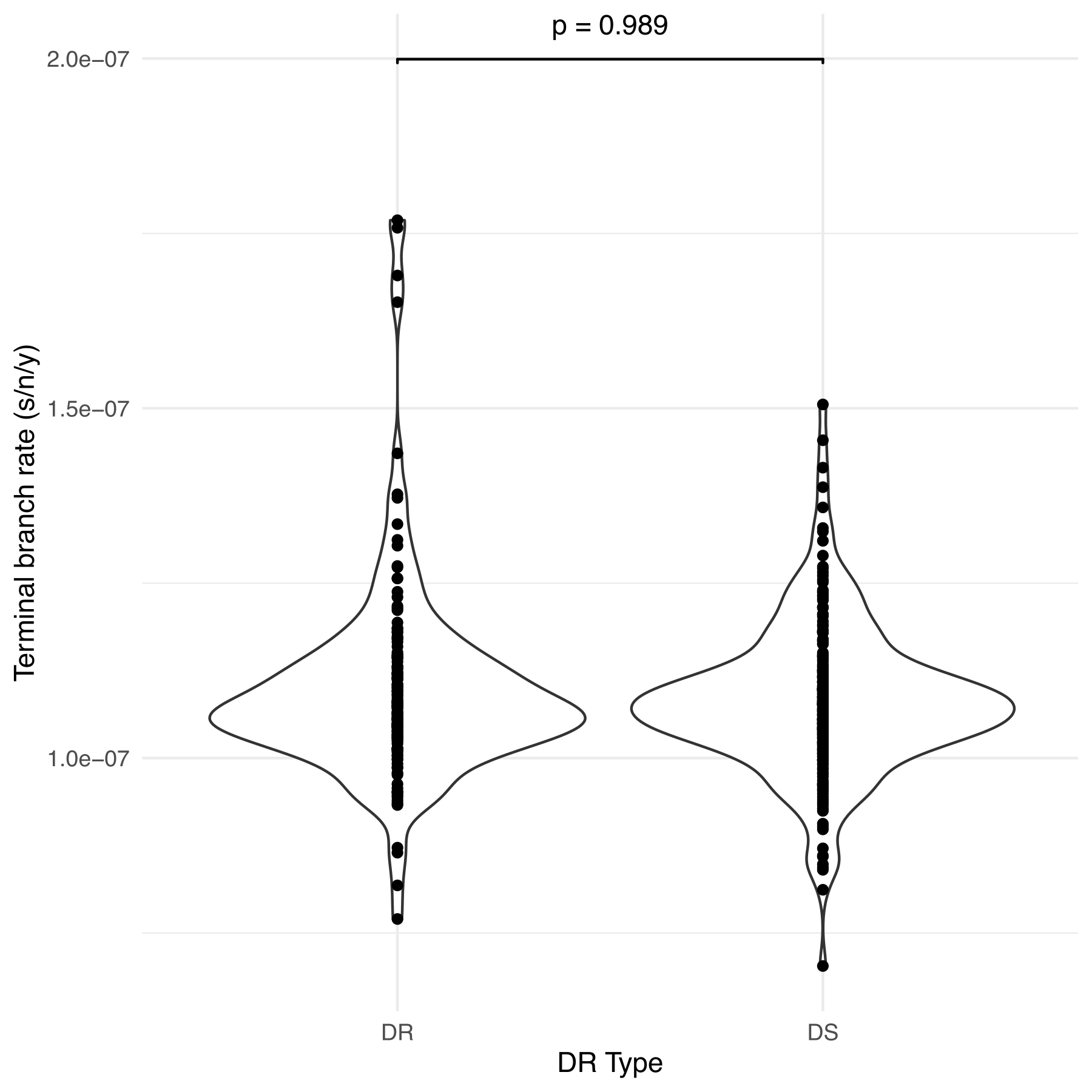


**Fig S3: Evolutionary rates on terminal branches leading to drug resistant (DR) and drug sensitive (DS) samples estimated under the uncorrelated lognormal relaxed clock model, the Bayesian Skygrid tree model, and the GTR+F+I nucleotide substitution model.** The difference was not statistically significant (Kolmogorov–Smirnov test: d = 0.0469, p =0.9894).


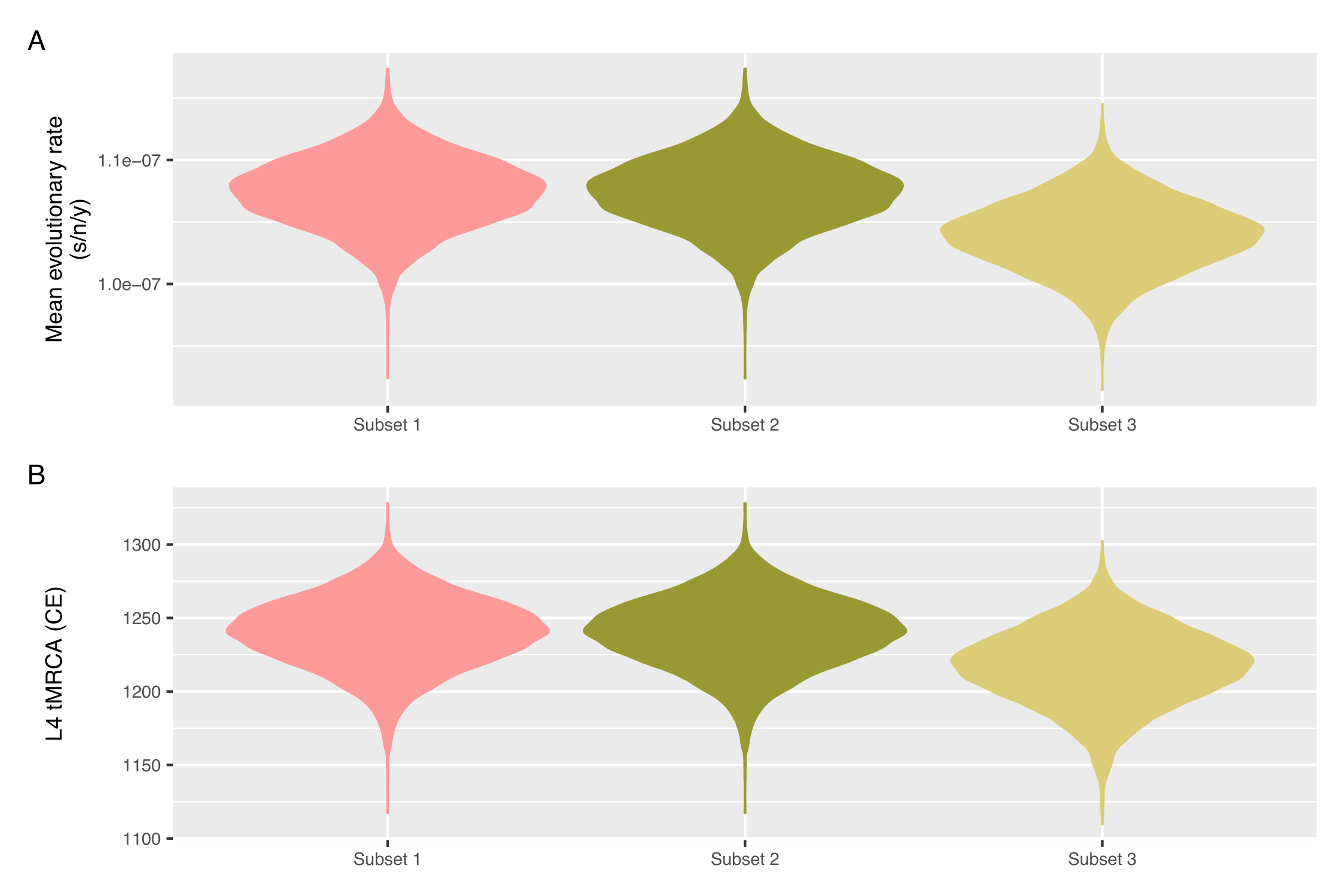


**Fig S4: Bayesian MTB L4’s evolutionary rates (A) and tMRCAs (B) estimated from three independently sub-sampled data sets (Table S2).**

**
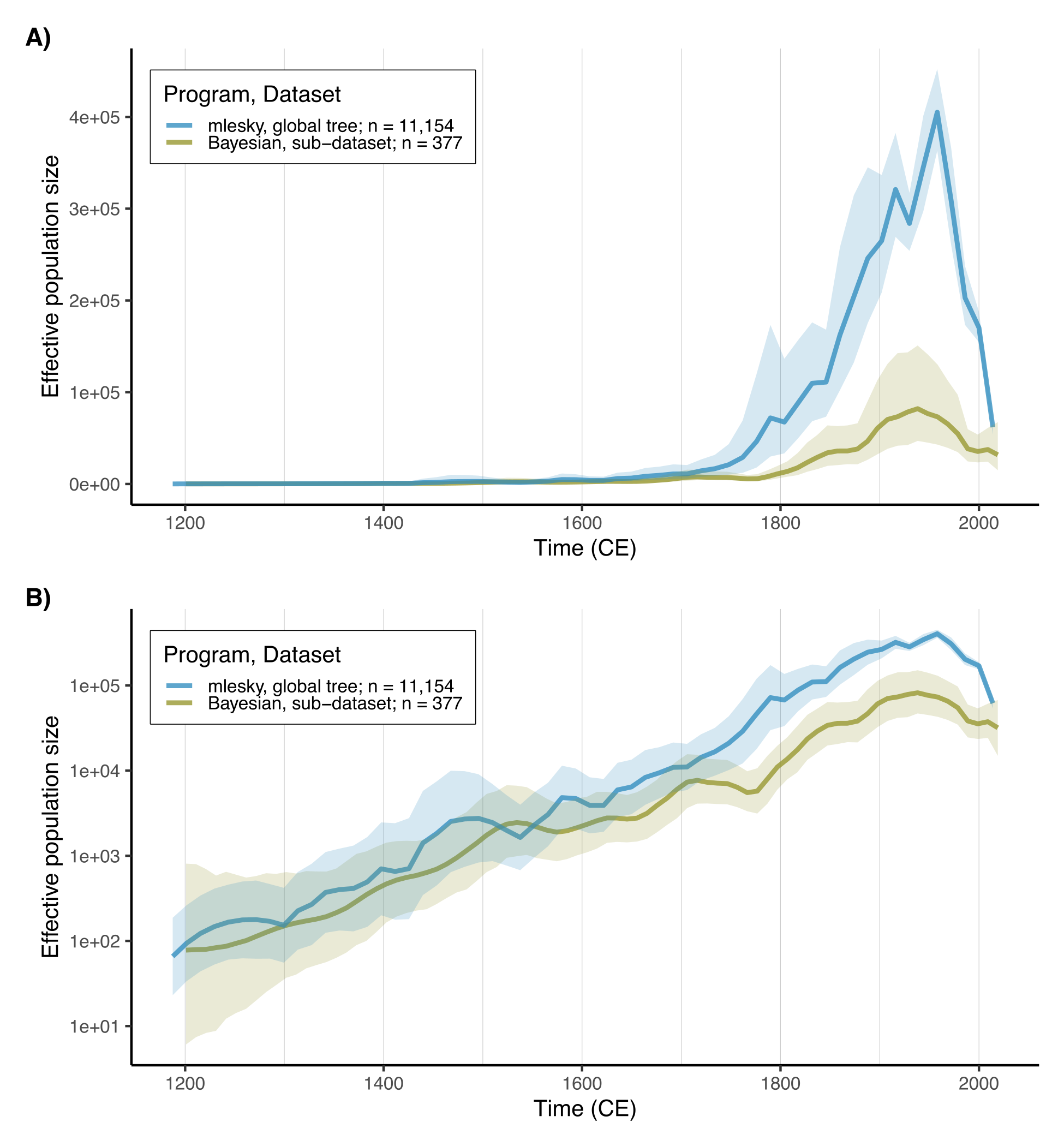
**

**Fig S5 Overall effective population size of MTB L4 shown on a linear scale (A), and on a log scale (B).** Blue lines present the result obtained from an analysis of the global time-calibrated MTB L4 phylogeny (Fig. 3) using the mlesky package in R. Green lines present the result obtained from the initial Bayesian tip-dating analysis of the down-sampled data set of 377 sequences by using BEAST v1.10.4 (6) with the strict clock and the Skygrid coalescent tree model.


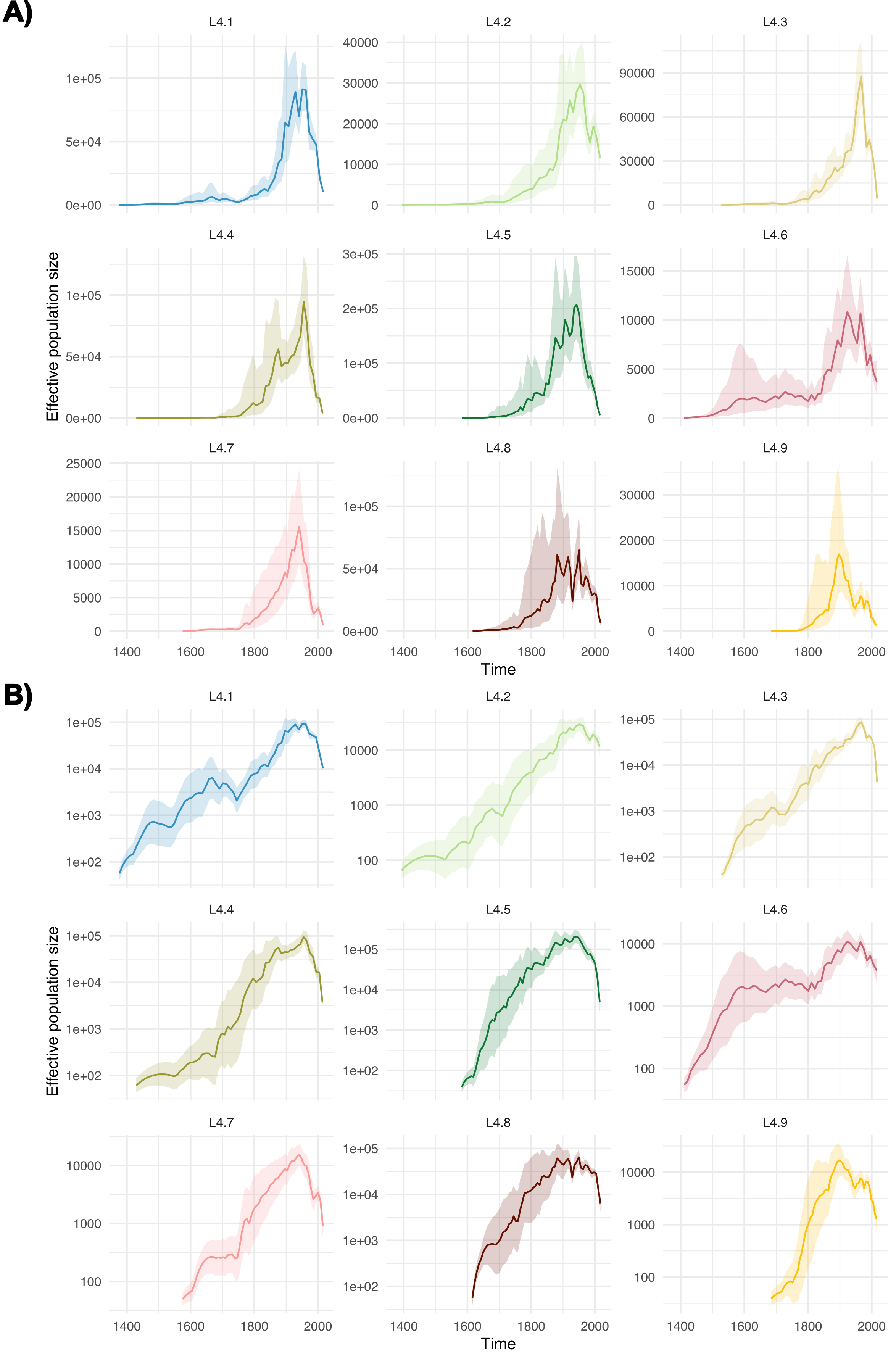


**Fig S6: Effective population sizes of the nine major MTB L4 sub-lineages shown on a linear scale (A), and on a log scale (B).**

# Supplementary tables

**Table S1. A complete list of samples analysed in this study.** Coverages and mean depths were computed by mapping reads to the H37Rv reference genome (NCBI’s RefSeq accession number: NC_000962.3). mtbtyper (<https://github.com/ythaworn/mtbtyper>) and TB-Profiler (2) were used to genotype and to determine the bacterial drug resistance type with default settings, respectively. The column “year_lsd2” contains tip dates used in the lsd2 tip dating analysis.

**Table S2. List of samples used in the Bayesian tip-dating analysis and model selection.** See legend to **Table S1**.

**Table S3. Clock and tree prior model selection analysis.** The models are sorted by log marginal likelihood scores from largest (best-fit) to smallest (poorest fit). Log Bayes factors were computed by finding the differences between the models’ log marginal likelihood scores.

**Table S4. AIC scores of the nine explored state transition and root prior models in the stochastic character mapping analysis.**

# References

1. Harshil Patel, Beber ME, Han DW, Nf-Core Bot, Manning J, Yates JAF, Espinosa-Carrasco J, Ewels P, Borry M, Domissy A, Ziff O, Jahdoos, Menden K. 2022. nf-core/fetchngs: nf-core/fetchngs v1.9 - Plutonium Prancer (1.9). Zenodo.

2. Phelan JE, O’Sullivan DM, Machado D, Ramos J, Oppong YEA, Campino S, O’Grady J, McNerney R, Hibberd ML, Viveiros M, Huggett JF, Clark TG. 2019. Integrating informatics tools and portable sequencing technology for rapid detection of resistance to anti-tuberculous drugs. Genome Med 11:41.

3. Fellows Yates JA, Lamnidis TC, Borry M, Andrades Valtueña A, Fagernäs Z, Clayton S, Garcia MU, Neukamm J, Peltzer A. 2021. Reproducible, portable, and efficient ancient genome reconstruction with nf-core/eager. PeerJ 9:e10947.

4. Sabin S, Herbig A, Vågene ÅJ, Ahlström T, Bozovic G, Arcini C, Kühnert D, Bos KI. 2020. A seventeenth-century *Mycobacterium tuberculosis* genome supports a Neolithic emergence of the Mycobacterium tuberculosis complex. Genome Biol 21:201.

5. Kay GL, Sergeant MJ, Zhou Z, Chan JZ-M, Millard A, Quick J, Szikossy I, Pap I, Spigelman M, Loman NJ, Achtman M, Donoghue HD, Pallen MJ. 2015. Eighteenth-century genomes show that mixed infections were common at time of peak tuberculosis in Europe. Nat Commun 6:6717.

6. Hill V, Baele G. 2019. Bayesian Estimation of Past Population Dynamics in BEAST 1.10 Using the Skygrid Coalescent Model. Mol Biol Evol 36:2620–2628.

7. Menardo F, Loiseau C, Brites D, Coscolla M, Gygli SM, Rutaihwa LK, Trauner A, Beisel C, Borrell S, Gagneux S. 2018. Treemmer: a tool to reduce large phylogenetic datasets with minimal loss of diversity. BMC Bioinformatics 19:164.

8. To T-H, Jung M, Lycett S, Gascuel O. 2016. Fast Dating Using Least-Squares Criteria and Algorithms. Syst Biol 65:82–97.

9. Aiewsakun P, Katzourakis A. 2015. Time dependency of foamy virus evolutionary rate estimates. BMC Evol Biol 15:119.

10. Arning N, Wilson DJ. 2020. The past, present and future of ancient bacterial DNA. Microb Genomics 6.

11. Kalyaanamoorthy S, Minh BQ, Wong TKF, Von Haeseler A, Jermiin LS. 2017. ModelFinder: Fast model selection for accurate phylogenetic estimates. Nat Methods 14:587–589.

12. Minh BQ, Schmidt HA, Chernomor O, Schrempf D, Woodhams MD, von Haeseler A, Lanfear R. 2020. IQ-TREE 2: New Models and Efficient Methods for Phylogenetic Inference in the Genomic Era. Mol Biol Evol 37:1530–1534.

13. Rambaut A, Drummond AJ, Xie D, Baele G, Suchard MA. 2018. Posterior Summarization in Bayesian Phylogenetics Using Tracer 1.7. Syst Biol 67:901–904.

14. Revell LJ. 2024. phytools 2.0: an updated R ecosystem for phylogenetic comparative methods (and other things). PeerJ 12:e16505.

15. R Core Team. 2020. R: A language and environment for statistical computing. R Foundation for Statistical Computing, Vienna.
